# Barriers and facilitators to improving the cascade of HIV care in Ontario: a mixed method study

**DOI:** 10.1186/s12913-023-10481-z

**Published:** 2024-01-10

**Authors:** Lawrence Mbuagbaw, Saranee Fernando, Chloe Lee, Maureen Owino, Cynthia Youssef, M. Elizabeth Snow

**Affiliations:** 1 Department of Health Research Methods, Evidence and Impact, McMaster University, 1280 Main Street West, Hamilton, ON L8S4L8 Canada; 2 Department of Anesthesia, McMaster University, Hamilton, ON Canada; 3 Department of Pediatrics, McMaster University, Hamilton, ON Canada; 4 Biostatistics Unit, Father Sean O’Sullivan Research Centre, St. Josep’s Healthcare, Hamilton, ON Canada; 5 Centre for Development of Best Practices in Health (CDBPH), Yaoundé Central Hospital, Yaoundé, Cameroon; 6 Division of Epidemiology and Biostatistics, Department of Global Health, Stellenbosch University, Cape Town, South Africa; 7 Centre for Advancing Health Outcomes, Vancouver, BC Canada; 8 School of Population & Public Health, University of British Columbia, Vancouver, BC Canada; 9 McMaster University, Bachelors of Health Sciences Program, Hamilton, ON Canada; 10 The Canadian HIV Trials Network (CTN), Vancouver, BC Canada; 11 Committee for Accessible AIDS Treatment, Toronto, ON Canada; 12 York University, Toronto, ON Canada

**Keywords:** HIV, Theoretical domains framework, Initiation, Adherence, Retention, Care Cascade, Ontario

## Abstract

**Background:**

Engagement in care is important for people living with HIV (PLH) to achieve optimal outcomes**.** Several strategies have been developed to improve client flow through the HIV care cascade, specifically targeting initiation of treatment, adherence to antiretroviral therapy (ART), retention in care, and engagement in care. We have previously identified effective care cascade strategies in a systematic review. Initiation of ART could be improved by mobile health interventions, and changes in healthcare delivery. Adherence to ART could be improved by mobile health interventions, incentives, counselling, and psychotherapy. Retention in care could be improved by mobile health interventions, incentives, education, and electronic interventions. The aim of this study was to investigate barriers and facilitators to implementing these effective interventions in HIV clinics in Ontario, Canada.

**Methods:**

We conducted a sequential explanatory mixed methods study. In the quantitative strand, we administered a survey to health workers who provide care to PLH to identify barriers and facilitators. In the qualitative strand, we conducted in-depth interviews informed by the theoretical domains framework (TDF) with health workers and with PLH to explain our quantitative findings. Qualitative and quantitative data were merged to create meta-inferences.

**Results:**

Twenty health workers from 8 clinics in 9 cities in Ontario took the survey. Nine PLH and 10 health workers participated in the qualitative interviews. Clinics in Ontario implemented all the effective interventions identified from the literature for initiation of treatment, adherence to ART, and retention in care despite concerns about resources. Barriers to physical and financial access to care, the workload for tailored care, and expertise were identified by both health workers and PLH. Key facilitators were virtual care and client preparedness through education and peer support.

**Conclusion:**

Clinics in Ontario appear to implement several evidence-based strategies to improve PLH engagement. There is a need for more health workers with skills to address unique PLH needs. Virtual care is beneficial to both health workers and PLH.

**Supplementary Information:**

The online version contains supplementary material available at 10.1186/s12913-023-10481-z.

## Background

Close to 37 million people are living with human immunodeficiency virus (HIV) worldwide [1], of which 71,000 reside in Canada [2] (amounting to over $92 billion in economic losses dues to healthcare, lost labour productivity, and quality of life) [3]. Even though the number of new infections is decreasing, the number of people with HIV is rising [4]. This is because they are living longer and healthier lives, mostly due to antiretroviral therapy (ART) [4].

It is recommended that people with HIV should start treatment immediately following their diagnosis. However, only about 80% of people diagnosed with HIV start treatment within 3 months of diagnosis in Ontario [5]. Initiating treatment is known to be affected by socioeconomic status, stigma, substance use, mental health, and disease severity [6–9]. Youth, people who inject drugs, and Black people often have lower rates of treatment initiation [10], therefore tailoring of interventions is required to address the needs of these groups of people at higher risk of initiating treatment late.

Despite being a pillar of HIV management, adherence to ART is often suboptimal [11]. This leads to worse treatment outcomes and greater costs (e.g., treatment switches, more hospitalizations and death) [12–14]. The highest levels of adherence are recommended to ensure optimal clinical and biological outcomes [15, 16]. Moreover, increased longevity of people with HIV implies they would take medication for longer, and therefore strategies should be in place to support adherence over the lifetime of the individual.

Retention in care is essential for PLH to receive social support, and clinical, and laboratory care. In a Canadian cohort, only 7.5% of people with HIV had a gap in care over 2 years (≥ year with no viral load), but up to 20% had discontinuous care (only 1 viral load/year in ≥ 1 year) [17]. If up to 20% of the 17,000 [18] people with HIV in Ontario have discontinuous care, more than 3000 people in Ontario with suboptimal care may be more likely to experience treatment failure, develop resistant strains, and transmit the virus.

The need to rethink HIV care strategies was recently highlighted in an American cost-effectiveness model indicating that improved linkage to care and retention (by 20% and 50% respectively) will reduce HIV incidence by 54% and mortality by 64% with a cost-effectiveness ratio of $45,300 per quality-adjusted life year (QALY) gained [19]. Several studies have developed interventions to improve adherence to ART and retention in care [20]. These interventions vary in nature and complexity, and it is unclear which of these interventions should be scaled up and how, especially in high-income settings.

A recent systematic review in World Health Organisation (WHO) stratum A (a list of countries, including Canada and the USA, with very low child mortality and low adult mortality) showed that many interventions were not effective on any outcomes or failed to impact both adherence and clinical outcomes [21]. On the other hand, a US-based study identified some effective interventions (for adherence alone) including interactive discussions, pager messages, and home visits [22]. Further, ten best practices for improving linkage and retention to care including case management and use of motivational interviewing were identified in a 2016 systematic review, although the authors noted that more rigorous study designs were needed to evaluate their effectiveness [23].

In 2020, we completed an overview of systematic reviews on interventions aimed at improving the care cascade. We found important knowledge gaps such as limited inclusion of vulnerable populations, poor quality of primary studies, and inadequate tailoring of interventions. We also compiled a list of effective interventions [24]. How this list maps unto what is currently done in Ontario is unknown.

The standard of HIV care in Ontario is based on the Ontario Clinical Care Guidelines for Adults and Adolescents Living with HIV in Ontario, Canada [25]. The goals are to provide effective long-term treatment, manage other health issues, address social determinants of health that might impact engagement in care and to improve quality of life. These guidelines describe specific approaches support initiation of HIV treatment, adherence to treatment and retention in care. There are additional population-specific considerations for women, men who have sex with men, Black populations and people using recreational drugs. These guidelines also encourage providing comprehensive HIV care using integrated models of care that involve an interdisciplinary team but note that not all providers will have access to the recommended services or skills [25]. While some countries are on track to meet the UNAIDS 95-95-95 targets for 2030 (95% of the PLH knowing their HIV status, 95% of the people who know their status are on ART, and 95% of PLH on ART virally suppressed) [26], many are not. This suggests that there is a disconnect between the available evidence, implementation of evidence and subsequent downstream outcomes. This study is aims to understand if interventions known to be effective are implemented in Ontario and the barriers and facilitators health workers and clients may face in applying these interventions.

The main objective of this study is to identify barriers and facilitators to the implementation of effective initiation-, adherence-, and retention-enhancing interventions in Ontario.

### Research paradigm and theoretical framework

Mixed methods combine qualitative and quantitative data to develop a richer body of evidence with sufficient depth and breadth to comprehensively address a given research question [27]. A pragmatist worldview was adopted to explore what works. The sampling, data collection, and interpretation were guided by the theoretical domains framework (TDF), an integrative, overarching, flexible framework of theories aimed at behaviour change [28]. It has been used successfully in the exploration of barriers and facilitators to implementing evidence-based behaviours [29]. It integrates several psychological theories, making it a flexible framework for behaviour change in diverse settings.

### Research questions

This work was informed by quantitative, qualitative, and mixed-methods research questions.

The quantitative research questions were:

- What interventions are implemented to improve outcomes in the HIV care cascade (initiation, adherence, retention)?
- Why are effective interventions not implemented?

The qualitative research question was:

- What are the barriers and facilitators to using effective interventions to improve the HIV care cascade?

The mixed-methods research question was:

- How do health workers and PLH perceive their ability to deliver or engage with interventions aimed at improving the HIV care cascade?

## Methods

### Patient and public involvement

The research questions addressed in this study are part of a program of research formulated and refined based on input from the Ontario HIV Treatment Network (OHTN), a non-profit network, as part of their strategy to close gaps in the cascade of care for key populations. People living with HIV contributed to the project as peer recruiters.

### Ethics

This study was approved by the Hamilton Integrated Research Ethics Board (HIREB), project # 7953.

### Design

We conducted a sequential explanatory mixed methods study (quan** → **QUAL) in HIV clinics in Ontario. This design comprised two phases: a quantitative cross-sectional electronic survey phase followed by an in-depth qualitative phase.

### Setting

This study was conducted in Ontario, the most populated province in Canada, where 42% of the People Living with HIV (PLH) in Canada reside [30]. Of the estimated 22,461 people living with HIV in Canada in 2020, only 89.0% were aware of their status, of which 86.7% were on ART [31]. The communities most affected are men who have sex with men, people who inject drugs, Black people, Indigenous People, and at-risk women such as single mothers [32].

### Sampling

In the *quantitative* phase, we developed a sampling frame from the mailing lists of the Ontario HIV Treatment Network (OHTN), and the HIV Outpatient Clinic Network (OCN) which includes 20 clinics. Our sampling frame included all of the target population i.e., HIV clinics in Ontario, and therefore no sample size was estimated. However, response rates were measured to assess if our sample is representative of HIV clinics in Ontario. Any staff or care providers working in an HIV clinic that provides care to PLH in Ontario were eligible to take the survey.

In the *qualitative* phase, we reached out to health workers who expressed interest in the survey in taking part in the in-depth interviews. Interested participants had to meet the following criteria: having taken part in the online survey, having at least 1-year experience in providing care to PLH, and being knowledgeable about initiation, adherence, or retention strategies. We worked with peer recruiters to purposefully identify a diverse group of PLHs receiving care in Ontario through their social and personal networks. To be eligible, participants were required to be above 16 years of age and living with HIV for more than 1 year.

### Data collection

In the *quantitative* phase, we conducted an electronic survey of HIV clinics in Ontario. The survey was designed according to expert recommendations for electronic surveys [33], and was developed for this study. We used RedCAP, a secure web application for building and managing online surveys and databases [34]. This survey targeted health workers at HIV clinics in the province of Ontario. According to the OHTN, there are 21 clinics across Ontario, with an average of 4 staff members who provide direct care per clinic. We elicited responses from physicians, physician assistants, nurses, social workers, counsellors, and pharmacists. We collected demographic information such as age (years), gender (man/woman/non-binary/cis/trans/other), profession (nominal), contact with PLH (yes/no; hours/week), duration of work with PLH (months), name of the clinic (nominal), and location of clinic (nominal). Participants were presented with a list of effective interventions identified in our overview of systematic reviews [24] and asked if these interventions are implemented or have been previously tried and abandoned in their clinics/HC (yes/no/don’t know). They were asked to identify which of the four domains of the Rating of Included Trials on the Efficacy–Effectiveness Spectrum (RITES) tool impeded the implementation of these interventions (yes/no/don’t know) (See Supplementary file 1). Participants were also asked if they were willing to take part in an in-depth interview.

In the *qualitative* phase, we interviewed both health workers and PLH residing in Ontario. Health workers who took the electronic survey and who were willing to participate were contacted to set up a convenient time for an interview. We planned to enroll PLH directly from the clinics, but the study was conducted during the COVID-19 pandemic and clinic attendance at that time was low. As such, we hired peer recruiters to identify PLH receiving care in Ontario. Interested people were referred to a qualitative interviewer, who provided them with study information forms and invited them to provide consent. The study objectives and procedures were explained to them. Interviews were conducted over the phone by a trained interviewer using a structured interview guide developed from the findings of the quantitative phase and the theoretical domains framework (TDF). Two interview guides were used in data collection, one for health workers and the other for PLH. Participants each received a $50 gift card.

Qualitative interviews occurred during the COVID-19 pandemic between July 2021 and August 2022 and included both groups (PLH and health workers) with overlapping interview timelines. Technical issues, such as audio recording challenges over the phone occurred during some of the interviews. It was simple to toggle between interviews due to the interview guides being mostly the same between groups, with minor modifications made to address differences between health workers and PLH. The interview guides covered effective interventions for improving the HIV care cascade, including Initiation: changes in healthcare delivery & mobile health; Adherence: psychotherapy, counselling, incentives, and mobile health; and Retention: incentive, electronic, mobile health, and education. Specifically, questions asked: What makes an intervention difficult to comply with (barriers)? And What makes an intervention easy to comply with (facilitators)? Twelve key domains were assessed, with questions probing under each domain. For example, under ‘Skills’, the interview guide asked, “Do you have the skills to comply with the intervention?” Questions were modified slightly for healthcare workers based on their specific profession.

Key issues such as experiences with initiation, adherence, retention strategies, and preferences were discussed. Interviews were conducted using plain language to enhance understanding of concepts. The interventions were described in detail in plain language to accommodate different levels of understanding. For interventions that were implemented at their clinic and those that were not, PLH and health workers were asked to comment on the factors that facilitate or impede their implementation. Participant responses were organized within each domain of the TDF.

### Data analysis

For the *quantitative* data, sociodemographic characteristics and responses were summarised as counts (percentages). We used tables to summarize the interventions implemented in at least one clinic, comparing them to interventions identified in the literature. The reasons why interventions were not implemented were summarized as counts.

*Qualitative* data were coded by two independent coders using the TDF as a coding framework (See Supplementary file 2) and the coding was compared for consistency. Content analysis was conducted using an open coding approach in Nvivo 12 software. Health workers and PLH data were analyzed separately using two different Nvivo 12 files. Two qualitative researchers were involved in the analysis. Both individuals read through the transcripts before performing analyses. An initial analysis was performed independently by one qualitative researcher (S.F.). Data was coded according to the TDF employing a deductive coding process. Codebooks were created for both clients and healthcare workers. The codebooks were discussed with the second qualitative researcher (M.E.S.) and disagreements in coding were resolved. A second cross-validation analysis was performed by the second qualitative researcher (M.E.S.) using five randomly selected transcripts from each participant group. Finally, another validation was performed by the primary qualitative researcher (S.F.). Content analysis was performed to generate descriptive findings of 19 transcripts (10 health professionals, 9 PLH).

To *integrate* our data we drew inferences from both qualitative and quantitative strands, and across strands, and conclusions were from these meta-inferences [35]. We have displayed our findings in a mixed methods figure to show patterns across the qualitative (between health works and PLH) and quantitative data. We focused on triangulating intervention efficacy with pragmatism (implementability), health worker perceptions of implementability, and client preference. When data do not converge, alternate theoretical frameworks were explored to explain divergence and used to generate new research questions [36].

## Results

### Quantitative

We emailed 19 clinics and received responses from 20 individuals in 8 clinics (response rate of 8/19 (42.1%) in 9 cities in Ontario, and included participants from Thunder Bay (1), Sudbury (1), Ottawa (2), Kingston (2), Whitby (1), Toronto (3), Hamilton (7), Guelph (1) and Windsor (1). We have outlined their characteristics in Table 1.

**Table 1:** Characteristics of participants in the quantitative phase

Variable	Statistic
**Age group (years)**	**n (%)**
21–30	1 (5)
31–40	3 (15)
41–50	5 (25)
51–60	8 (40)
> 60	3 (15)
**Gender**	**n (%)**
Female	15 (75)
Male	5 (25)
**Number of years providing HIV care**	**n (%)**
Up to 5 years	6 (30)
More than 5 years	14 (70)
**Role in HIV care**	**n (%)**
Physician	6 (30)
Nurse	6 (30)
Pharmacist	2 (10)
^a^Other	6 (30)
**Key populations seen in routine care**	**n (%)**
Indigenous people	13 (65)
People in the sex work industry	15 (75)
Adolescents and young adults	17 (85)
Women	18 (90)
Pregnant women	17 (85)
Men who have sex with men	18 (90)
People who use substances	18 (90)
Heterosexual men	18 (90)
People with mental health issues	18 (90)
Black people	20 (100)
Immigrants, refugees, and people without status	20 (100)

^a^Research, Director of clinical services, Clinical Nurse Coordinator, Clinic Manager/Nurse, Clinical Coordinator, Social worker (one of each)

All the effective interventions identified from the literature were implemented in at least one clinic, such as mobile health interventions and changes in healthcare delivery for initiation of treatment; mobile health, incentives, counselling, and psychotherapy for adherence to ART; and mobile health, incentives, education, and electronic interventions for retention in care. These are summarised in Table 2, where green indicates a match between the literature and implementation practices, and yellow indicates a mismatch between the literature and implementation practices. For the most part, the mismatches were interventions implemented in clinics that did not seem to be effective based on our systematic review [24].

**Table 2:**
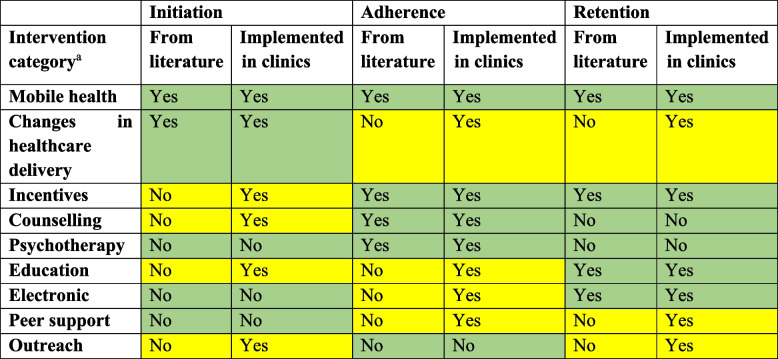
Summary of intervention types identified in the literature versus those implemented in clinics in Ontario

^a^Education: flyers, text, sessions; Mobile health: phone calls, text messages, app-based; Counselling: group or one on one sessions; Electronic: computer-based, interactive; Changes in health care delivery: change in the number of pills, the place where medication is delivered, dedicated staff or space etc.; Incentives: food, money, vouchers; Peer navigation or support: another person with HIV helping; Psychotherapy: cognitive behavioural therapy, motivational interviewing; Outreach: going to meet/find people in their communities

Green: Match between the literature and implementation practices

Yellow: Mismatch between the literature and implementation practices

**Fig. 1:**
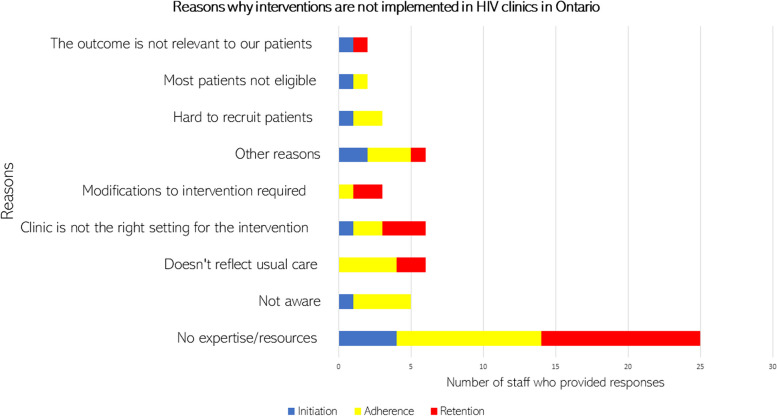
Reasons for not implementing effective interventions in HIV clinics in Ontario

Most often, the reason why some effective interventions were not implemented was related to a lack of expertise or resources. All the reasons, stratified by care cascade outcome, are shown in Fig. 1.

### Qualitative

Nine (9) PLH and 10 health workers were interviewed. The mean (standard deviation [SD]) age of the PLH was 42 (9.82). Three (3) were male, five (5) were female, and one (1) was gender fluid. The mean (SD) age of the health workers was 49.7 (9.42) years. One was male and 9 were female. Professions included physicians (4), nurses (3), nurse coordinators (2) and a pharmacist.

#### Barriers for PLH

Participants described multiple overlapping barriers when receiving care along the HIV Cascade of Care. For example, one participant, a 45-year-old woman living with HIV, described challenges related to language barriers, lack of privacy, and side effects of medication when asked about difficulties complying with HIV medication:

*“When you are in the shelter you don’t have privacy and you may not take medication as your status may be disclosed. The language barrier … they need someone to take them to the doctor. They need an interpreter. Not having an interpreter makes it difficult to go. Not taking medication and other people in the house can prevent adherence. Pressure from the family getting to know the [HIV] status. Some side effects of the medication. I don’t want to say the medication will change my body. If people see me then they know.”*

In some cases, medication adherence was difficult due to the size of antiretroviral pills, side effects, and the need for consistency when taking medications. Such factors led to lowered adherence to antiretroviral medications.

The need for privacy was highlighted, particularly in shared housing conditions where a person’s HIV status may be at risk of disclosure. Additionally, mental health conditions, particularly depression, were described as a potential barrier to feeling motivated to adhere to HIV medication. Reaching out for help was also difficult when the PLH was experiencing depression. Participants also described stigma and discrimination as a deterrent to care, as illustrated by a 42-year-old man:

*“Stigma is a major deterrent to care, because when someone sees you, for example, if someone sees you actually trying to access a program, trying to, or perhaps get medication or something, that person is going to come to your mind, oh this person is sick.”*

When participants required in-person care, access to care was sometimes challenging due to transportation issues. In one case, long trips to access care by using public transportation made it difficult to arrive at appointments on time:

*“But most of the programs they’re like Downtown which is like – it takes some time to get where you’re supposed to be on time… That was last week I had challenges with [unintelligible] of which I was outside I think for an hour to get the transport. And then like it takes another hour to get to where I was supposed to go.”* - 42-year-old woman

When describing the HIV care cascade, participants indicated the need for enhanced patient-oriented care, including individualized and specialized care depending on the needs of the patient. As one 42-year-old man articulated:

*“the resources that are missing, well to be honest, I would say a more individualised or specialised … not generalizing the care, like hoping that “OK now that this works”, so and so, it should work for every Black person in this category of this age.”*

This involved health care workers understanding what works for their patients and tailoring health care programming and medication more appropriately:

*“Like, I wouldn’t say it’s a barrier I would say it’s [pause] well it could be a barrier but what I think is not being able to get a doctor who truly understand(s) where you’re coming from in terms of what can and what doesn’t work for you personally, yeah. When the doctor is using more of their expertise…that’s how your body feels, and what you complain about how you feel, you know what I mean?”*- 40-50-year-old woman

During the COVID-19 pandemic, virtual appointments became a necessary mode of health care delivery. Though participants described the benefits of receiving virtual health care from the comfort of their homes, one 40–50-year-old woman described challenges with presenting the doctor certain physical ailments, such as wounds: “*I’m telling you I have a wound, it’s different from when you see me [in person] and how the big the wound is …”.*

#### Facilitators for PLH

The HIV cascade of care fostered a sense of community and belonging for PLH in Ontario. The peer support elements were described as “very important” to help participants “adhere to medication” through reminders and continued engagement. These community networks created a supportive environment, a sense of connection, and culturally inclusive community memberships. As one 42-year-old woman highlighted:

*“And sometimes we did like we encourage each other, we teach each other [unintelligible] how I cope or how I do, so like we share ideas.”*

In addition to supportive peer networks, participants described healthcare workers as helpful and essential for helping them navigate the programs, receive educational information, and accommodate their needs. As described by a 42-year-old man:

“I think they were the ones who kind of helped me grow, and grow, and grow, and grow. Because without their intervention and without their programs educating me, I wouldn’t be able to, being able to navigate myself throughout all these ordeals.”

Education allowed participants to feel more confident, as illustrated by one 42-year-old woman:

*“Through the knowledge and information that, a program that they have through that organisation, it makes me feel more confident and comfortable being, having that discussion and being, having a conversation from one who perhaps wouldn’t know what to ask.”*

#### Barriers for health workers

Health care workers described several barriers to providing comprehensive care due to resource limitations, namely, the need for more time and additional staff members. Limited time with patients did not allow for patient-provider relationship building. As one 52-year-old physician highlighted:

*“Like probably the big limitation is time and then – even this one patient, you know, I’ve seen her once, so I don’t know how much of a strong bond that is.”*

Additionally, health workers indicated the burden of extending their regular work roles to accommodate the needs of patients. One 32-year-old physician indicated the need for additional staff and resources to engage in essential tasks to ensure patient engagement:

*“but some of the things are easy to implement because they’re just things that you’d do in your care of the patients. Other things really require that you have staff that can reach out to patients, follow up with them, answer messages, which is more challenging too. Then you have to kind of be able to be spending money and other stuff, right? Like they have to be paying for the time with money.”*

Inadequate staffing would lead to the stretching of roles to accommodate patient needs. As illustrated by one 53-year-old nurse:

*“so, we used to have team meetings but we do not anymore as things got really hectic and our staffing numbers went down and so we were just basically functioning on a daily basis, day-to-day basis. So currently I am not involved in much in the clinical decision-making.”*

Health workers also described the need for patients to be invested in their care to enable the success of the HIV Cascade of Care as expressed by one 50-year-old clinical nurse coordinator:

*“Well you get a little cynical over time. And you know, interventions are one step in the process. The patient has to want to – you know, or when you're thinking of the three domains you talked about initially the patient has to collaborate or want to adhere to treatment. You can't – you know, you can get them to clinic and you can get them their meds, but you can't necessarily get them to take them all the time either.”*

Other primary barriers to care were related to access, namely the ability of patients to attend appointments and receive medication coverage in Ontario. Appointment attendance was impacted by several factors, including cell phone access, transportation, and mental health issues. Unfortunately, some patients were not accessible by phone during a time when virtual visits were the primary modality of care provided during the early part of the COVID-19 pandemic. One 53-year-old nurse described difficulties when trying to bring a patient to their appointment:

*“A couple of weeks ago we had a patient who lives in the [name] area which is about an hour from here and he is very marginalized, and he did not show up for his appointment here. However, there was follow-up and apparently the taxi cab said that they picked him up and brought him to [name] and dropped him at the hospital, but he never showed up to his appointment. So, either the patient has mental health or something was happening that day that he didn’t recall. So, we actually need someone for those really marginalized patients to actually bring them into clinic.”*

In Ontario, lack of complete HIV medication coverage “is a huge struggle for us”, impacting initiation to treatment. As one 32-year-old physician indicated: *“well I think if we had Pharmacare or, you know, the full access to antiretrovirals like some of the provinces, I think that would be less of a barrier. People do get into those problems, and they have financial sort of burdens which don’t always get disclosed to us and that being a barrier for them to stay on medication and keep up therapy.”*

This was particularly challenging for immigrants, who would find it difficult to navigate the system, thus impacting their treatment adherence. Immigrants can receive compassionate coverage and are required to enrol in appraisal programs to receive additional coverage. Challenges with compassionate coverage occurred for patients requiring multiple different medications:

“So, the barrier in that is if they have a genotype that is [unintelligible] in it, then with that's where the problem comes in to get compassionate coverage because often they'll need to be on two, three, four different medications from all different drug companies and not all of them have the compassionate program. So, if we can get them on a one pill once a day regimen then usually we can get coverage for them.” – 54-year-old physician

Finally, one participant described a deficiency in expertise and knowledge in new healthcare providers compared to those who have been providing care for PLH for decades. As illustrated by a 53-year-old nurse coordinator:

*“They [new hires] don’t have the same knowledge, OK. What I find is that it’s the transfer of knowledge and the other services... so that’s going to be an issue. My social worker will retire soon, and I have new staff and they don’t have quite the same knowledge and I find that’s where we need to do something and that’s the biggest fear for HIV future.”*

#### Facilitators for health workers

Health workers noted the importance of investing in optimizing patient health along the HIV cascade of care to promote successful initiation, treatment, and retention. This process involved flexible service provision from multiple providers along the patient care trajectory, education, and discussions surrounding medication adherence. Having these mechanisms in place was essential for the success of the program. As illustrated by a 53-year-old nurse:

*“Typically, when we start patients off just talking to the patients, you know, if they hear from a couple of different sources before they even start, you know, prescription medications it always is – it kind of sets things up to be successful. And after they, and sometimes even before, like we will see our patients and if they’re a new patient to the clinic nursing and social work will see them prior to them coming in a for a physician visit. So, at that initial visit we do discuss with the patient what our goals are here in this clinic and how they relate to HIV and what kind of management we use, so by management I mean using medications, what the expectations of the medications are in terms of frequency and dosing and adherence. And then we do some education, I do some education about the natural history of HIV and also how the medications will affect that and improve their outcomes in the long term.”*

Collaborative care was seen as essential for the delivery of comprehensive support due to the complex needs of PLH, including housing and social support. One participant described the benefits of partnering with an AIDS service organization to enable enhanced support for PLH:

*“So, our clinic is quite unique. We are one of two in all of Ontario that are [partnering] with an AIDS service organization. So, we rely heavily on the support workers to network and case manage with the nurses. So, they help us get…they connect people to local services for housing. They fill out paperwork for the Ontario Disabilities Support Program. They get people connected to the drug plan. All of those kinds of things. So, they help immensely with getting folks started.”* - 54-year-old physician

In instances where medication coverage was a problem, health workers endeavour to find solutions to help PLH access timely care: *“I think, you know, we try to do as much as we can to inform individuals to contact us if there is an issue with financial coverage of medications and we try to address those at every clinic. So, I think when we’re notified of them, we are trying to, you know, make that as seamless as possible, but that requires somebody to inform us that there is a problem”*- 48-year-old pharmacist.

Patient compliance was also noted as necessary for successful adherence to the HIV cascade of care. As health workers invested in multiple components of the program, patients were encouraged to invest time and energy into their appointments:

*“Because they’re all hospital employees, we’re very dependent on people coming to the clinic and getting that service, you know. Like we encourage them to come, but if they choose not to come, they’re sort of on their own.”*- 52-year-old physician

Virtual clinics were useful for patients who had access to phones. In these cases, patients did not require transportation, which would often involve hours of travel time. Despite the convenience of virtual visits, drawbacks included patients having to describe their health issues over the phone:

*“We used to go (a) five hour drive away to get them down in person, but now we can see people virtually or on the phone on the diagnosis so that has been helpful. Limitations from online consults, don’t have face to face communications, your assessment happens behind the telephone. What’s on their bodies they have to describe it on the phone and it’s hard to see it. They have access to internet, and email it to us. Some of our patients are marginalized and don’t have phones so it’s more of a challenge.”* - 50-year-old nurse

#### Impacts of the response to the COVID-19 pandemic

The response to the COVID-19 pandemic increased barriers to adherence along the HIV cascade of care. Participants described service disruption where “a lot of services had to stop”. Additionally, it took some time to establish virtual visits, creating a lag in the provision of essential care. Once virtual visits were established, though physicians preferred virtual visits, not all PLHs connected to the clinics had access to phones or computers. Therefore, loss to follow-up occurred among the most marginalized patients due to lack of access to the appropriate technology for virtual visits, compounded by mental health issues, and other life stressors. In the home setting, the lack of privacy was highlighted as a barrier to engaging in virtual care due to the presence of other family members within the home setting.

*“It’s been 2.5 years since COVID started. A lot of services had to stop. No face-to-face. It took some time before we had meetings online and even with the meetings, not a lot of people have privacy at home.”* - 45-year-old woman

### Data integration

**Fig. 2:**
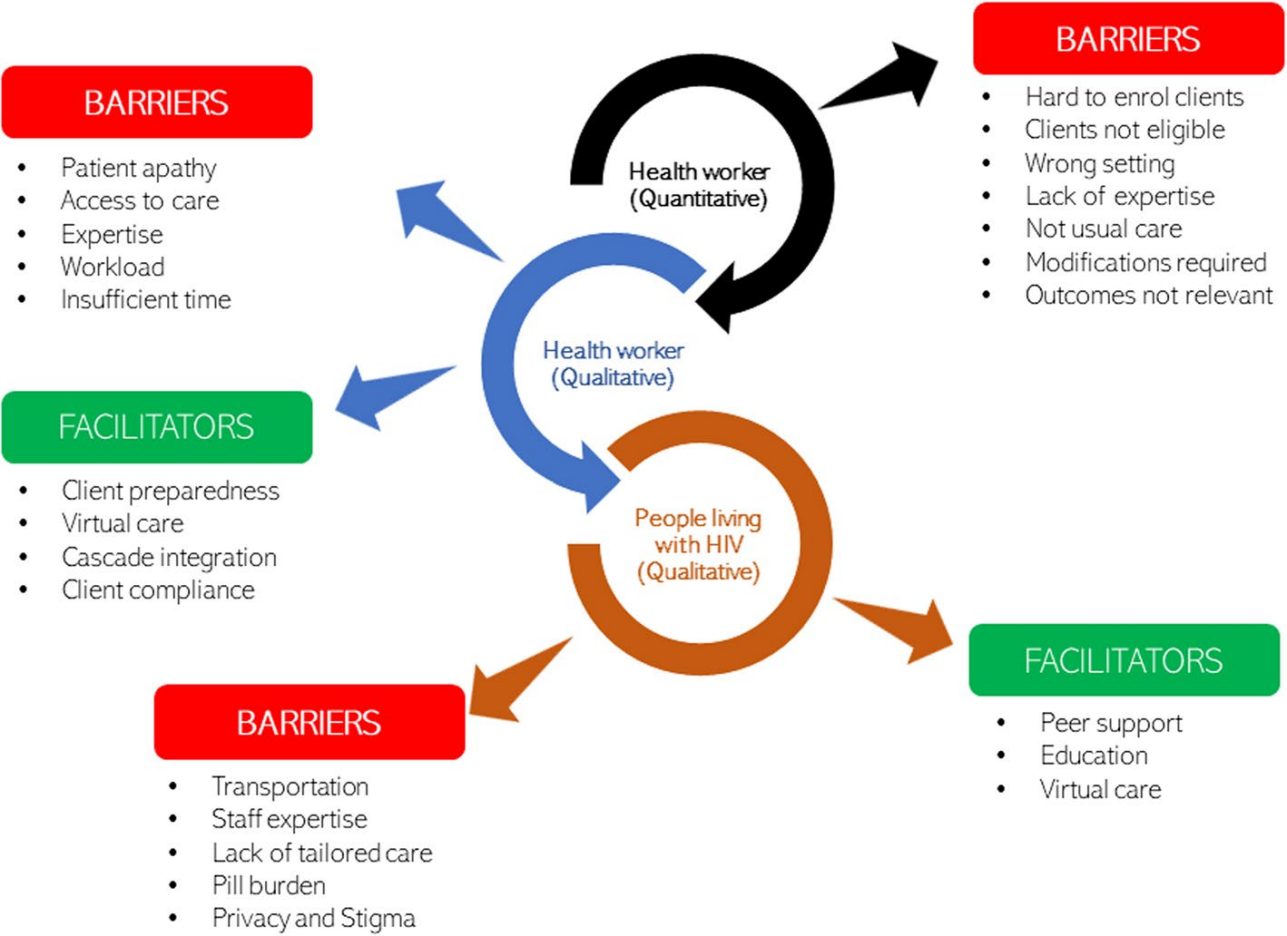
Linkages between qualitative and quantitative strands

While the quantitative strand included only health workers, the qualitative strand included both health workers and people living with HIV. Figure 2 shows the linkages between the qualitative and the quantitative strands and between health worker and PLH perspectives on barriers and facilitators.

#### Health workers and people living with HIV

Both health workers and PLH acknowledged limitations in access to care as important barriers to engaging with the HIV care cascade. Health workers noted access to specific medications, while PLH noted transportation issues. While these are different aspects of access to care, they both compromise continuity of care. Expertise in delivering specific interventions was identified by health workers and PLH. We found linkages between clients’ needs for individualized or tailored care match well with health worker reports of lack of expertise, time, and heavy workloads that precluded the provision of tailored care. Concerning facilitators, health workers found that client preparedness for care was an important part of the engagement, which could be facilitated by peer support and education on the PLH side. Likewise, virtual care was noted by both groups to be a facilitator. Some responses did not triangulate. For example, PLH noted pill burden and stigma to be important barriers, whereas health workers did not identify these as barriers. On the other hand, health workers noted patient apathy to be a barrier, which was not noted by PLH. Health workers also felt that integration of the whole cascade of care and patient compliance were facilitators.

#### Quantitative and qualitative

Lack of expertise was noted in both qualitative and quantitative strands as a barrier to the implementation of care cascade interventions. Several other concerns related to the clients in care (hard to enrol, not eligible, outcomes not relevant), and the nature of the intervention (inappropriate setting, modifications required, not usual care) speak to the disconnect between research and real-world practice, i.e., interventions that were found to be effective in the literature were not always pragmatic and easy to implement in the real world.

## Discussion

### Summary of findings

In this mixed-methods study, we have identified barriers and facilitators to engaging in the HIV care cascade in Ontario. Overall, from the health worker's perspective, skills, resources, and time are important assets required to enhance the care cascade. This would allow health workers to use a variety of strategies and meet the needs of clients for more tailored care. Expanding access to medication, and transportation to care is also a concern. PLH perceived peer support and education as facilitators to engaging in care. Virtual care, as experienced during the COVID-19 pandemic, has the potential to overcome some barriers.

The qualitative strand explains some aspects of the quantitative strand. For example, the lack of expertise to implement interventions was reported in both strands and by both health workers and PLH. When interventions require modifications, do not match the setting of care, do not fit in the ecology of usual care, are designed for outcomes not relevant to clients, or are designed such that many clients are not eligible, tailored care is needed. PLH specifically noted that some strategies for engagement in the care cascade did not “fit” them.

In the qualitative strand, we identified several additional barriers (sub-optimal client compliance, challenges in integrating all aspects of the care cascade, stigma and pill burden) not identified in the quantitative strand. This is not unusual, given the nature of the inquiry, allowing for more in-depth responses.

#### Expertise, resources, and time

While the current clinical guidelines encourage the provision of integrated care using interdisciplinary teams, they acknowledge that not all providers would have the skills and resources required within their networks to provide the breadth of care that some clients may need [25].

PLH receive care from a range of health workers including physicians, nurses, pharmacists, community health workers and others. Even within this diverse group of health workers, expertise certain skills may be lacking. Lack of expertise for specific interventions was a barrier noted in both the qualitative and quantitative strands among health workers and PLH alike. If we were to take the example of counselling and psychotherapy which have both been shown to improve adherence to antiretroviral therapy, and are implemented in some clinics in Ontario, this would only be possible if the staff had training in these techniques, and their workload and schedules allowed them to deliver enough sessions to have an impact. This is unlikely to be the case in high-burden clinics and could explain why workload and insufficient time are barriers noted by health workers. Other Canadian studies have reported physicians’ barriers to providing optimal counselling for HIV and other sexually transmitted diseases [37].

#### Mismatch between research evidence and real-world practice

In the quantitative strand, health workers noted specific barriers related to the lack of pragmatism of interventions reported in the literature. Several other studies have described the lack of generalisability of evidence from randomized trials. This occurs in part due to highly stringent inclusion criteria in trials and interventions that are not similar or do not fit with how care is delivered. This will in turn make it hard for providers to find eligible clients who are interested in the intervention. Further, it might be necessary to modify the intervention to make it applicable. One example would be an mHealth intervention that uses a dedicated platform to deliver motivational or educational text messages to clients to support adherence and retention in care. At a clinic, one would require a fully programmed computer and dedicated staff to enter client details and to monitor delivery of messages. Clients may not want to receive additional communication from their clinics, especially if they have concerns about privacy and stigma. Managing the text messaging platform may also be addition work for already overburdened staff. This in part speaks to the level of pragmatism of trials included in the systematic reviews. In the CASCADE database – a collection of 298 trials that been used to improve the HIV care cascade [20], only 80 (60%) were rated as pragmatic, based on the RITES tool.

One other barrier which health workers raised is that the outcomes targeted in the effective interventions may not be relevant to their client base. This is true for clinics that receive clients who have already initiated ART and therefore are only involved in the subsequent parts of the care cascade (adherence and retention).

Worth mentioning is that some interventions were implemented in some clinics despite not being supported by the evidence in our systematic review. There are some potential reasons for this. First, if clinics truly apply an integrated HIV care cascade model, then interventions may be applied across the whole spectrum, even though they only have impact on specific outcomes. For example, educational interventions which improve retention in care may be introduced at initiation of ART and continued throughout the course of care. Likewise, outreach interventions that enhance initiation of ART and retention in care would still be applied to encourage adherence throughout the course of care. Second, it is also possible that certain interventions, though not effective in the broader populations included in the systematic review, might be effective in a locoregional context after modification or tailoring. For example, while broadly speaking, incentives are not effective in enhancing initiation of ART, a specific incentive (e.g., cash or transport reimbursement) could help overcome transportation difficulties and encourage initiation of ART.

#### Strong points

Despite these challenges, virtual care stood out as a facilitator for both health workers and clients. Virtual care not only circumvents the need for transportation, but also may afford clients with some level of privacy that may encourage engagement in care. Further, for immunocompromised people living through a pandemic, virtual care would also limit their exposure to transmission. For the purposes of understanding the different points at which PLH engage in care the HIV care cascade is divided into initiation, adherence, and retention. In practice, integrating all the components of the HIV cascade makes sense since several interventions are effective on more than on component. Cascade integration was found to be a facilitator among health workers.

Further, individual level facilitators such client preparedness for HIV care and compliance with care identified by health workers are in line with the use of peer support and education reported by clients.

Our findings, put together suggest that clinics differ in important ways (resources, expertise, scope of care) that have an impact on the HIV care cascade. This is not unusual, given that it is unlikely that staffing, proximity to community resources, access to specialised services and even the distance patients must travel will be uniform across clinics. Further work could investigate the minimum capacity required for HIV clinics to deliver evidence-based holistic and interdisciplinary care.

This study is not without limitations. In the quantitative phase, responses to the electronic survey were suboptimal, as the survey started in the middle of the first wave of the COVID-19 pandemic when health workers were adjusting to virtual care. While we did not reach the numbers we were hoping for, we covered 9 of the main cities in Ontario and achieved a clinic response rate of 42.1%, which is close to the average for electronic surveys [38]. In the qualitative strand, we faced some difficulty in building trust over the phone for PLH interviews during the pandemic using a non-peer researcher (peer interviews may be more successful in eliciting in-depth responses from PLH). Technical difficulties compromised the audio quality in some interviews. The main strengths of this study are the robust methodology - using both qualitative and quantitative approaches, the use of the TDF to elucidate barriers and facilitators and the merging of dual perspectives from health workers and clients.

## Conclusion

The HIV Clinics in Ontario included in this study reported implementation of several evidence-based strategies to improve PLH engagement in the HIV care cascade. Further commitments are needed to enhance the skills and resources available to health workers to provide adapted care for PLH. Peer support, education, and virtual care are valuable in enhancing client-side engagement.

### Supplementary Information

**Additional file 1.** Electronic Survey.

**Additional file 2.** PLH Sample Responses.

## Data Availability

The datasets used and/or analysed during the current study available from the corresponding author on reasonable request.

## Abbreviations

AOHC: Association of Ontario Health Centres
ART: Antiretroviral Therapy
HW: Health Worker
OHTN: Ontario HIV Treatment Network
PLH: People Living with HIV
TDF: Theoretical Domains Framework
