# Supplementary material for: Barriers and facilitators to improving the cascade of HIV care in Ontario: a mixed method study
Source: BMC Health Serv Res. 2024 Jan 10;24:48. doi: 10.1186/s12913-023-10481-z (PMC10782539; doi:10.1186/s12913-023-10481-z)
Supplement: Supplementary file 1 — Additional file 1. Electronic Survey. [file 12913_2023_10481_MOESM1_ESM.pdf]

Electronic Survey

Record ID

____

What is your role?

- ○ Physician
- ○ Physician Assistant
- ○ Nurse
- ○ Social worker
- ○ Counsellor
- ○ Pharmacist
- ○ Nutritionist
- ○ Outreach worker
- ○ Clinical Coordinator
- ○ Other

If other, name your role.

____

How many employees are at your clinic/ health centre ?

____

Do you provide care to any of these groups of people at your clinic/ health centre? (Select all that apply)

- ☐ Women
- ☐ Pregnant women
- ☐ Adolescents and young adults (< 35 years-old)
- ☐ Men who have sex with men
- ☐ Substance users (injection and non-injection drug users)
- ☐ Heterosexual men
- ☐ People with mental health concerns
- ☐ African, Caribbean or Black people
- ☐ Sex workers
- ☐ People with mental health issues
- ☐ Immigrant, refugees and non-status people

Do you have in-person contact with people with HIV?

- ○ Yes
- ○ No
- ○ Do not know

How long have you been providing care for HIV ?

- ○ less than 1 year
- ○ about 1-2 years
- ○ about 2-3 years
- ○ about 3-4 years
- ○ about 4-5 years
- ○ more than 5 years

In what city is your clinic/ health centre located

____

What is the name of your clinic/ health centre?

____

About initiating antiretroviral therapy (starting treatment for the first time):

The following interventions have been found to be effective in improving initiation of antiretroviral therapy. Which of these interventions are currently implemented in your clinic/ health centre?

	Yes	No	Don't Know
Intervention 1	○	○	○
Intervention 2	○	○	○
Intervention 3	○	○	○

Which of the following reasons may explain why this intervention is not implemented?

- ☐ I am not aware of this intervention
- ☐ Most patients are not eligible for this intervention
- ☐ It will be hard to recruit patient to take part in the intervention
- ☐ My clinic/ health centre is not the right setting for this intervention
- ☐ We don't have the expertise and resources (space, money equipment, etc.,) to deliver this intervention
- ☐ We might have to modify the delivery of the intervention to be able to us it
- ☐ We might have to modify the intervention to ensure that patients are compliant
- ☐ It requires very close follow up
- ☐ The outcome of this intervention is or relevant to participants
- ☐ It doesn't reflect usual care
- ☐ Other reasons (please explain below)

Do you implement any interventions to improve initiation to ART in your clinic that are not mentioned above?

○ Yes ○ No ○ Don't Know

If yes describe the intervention(s):

____

If other, please explain

____

Are there any interventions to improve adherence to antiretroviral therapy that have been tried and abandoned in your clinic/ health centre

○ Yes ○ No ○ Don't Know

If yes, please describe the intervention(s) and why it was abandoned.

____

About adherence (taking medication as prescribed) to antiretroviral therapy:

The following interventions have been found to be effective in improving adherence to antiretroviral therapy. Which of these interventions are currently implemented in you clinic/ health centre?

	Yes	No	Don't Know
Interventions 1	◯	◯	◯
Int. 2	◯	◯	◯
Int. 3	◯	◯	◯

Which of the following reasons may explain why this intervention is not implemented?

- ☐ I am not aware of this intervention
- ☐ Most patients are not eligible for this intervention
- ☐ It will be hard to recruit patient to take part in the intervention
- ☐ My clinic/ health centre is not the right setting for this intervention
- ☐ We don't have the expertise and resources (space, money equipment, etc.,) to deliver this intervention
- ☐ We might have to modify the delivery of the intervention to be able to us it
- ☐ We might have to modify the intervention to ensure that patients are compliant
- ☐ It requires very close follow up
- ☐ The outcome of this intervention is or relevant to participants
- ☐ It doesn't reflect usual care
- ☐ Other reasons (please explain below)

Do you implement any interventions to improve adherence to ART in your clinic that are not mentioned above?

◯ Yes ◯ No ◯ Don't Know

If yes describe the intervention(s):

____

If other, please explain

____

Are there any interventions to improve adherence to antiretroviral therapy that have been tried and abandoned in your clinic/ health centre

◯ Yes ◯ No ◯ Don't Know

If yes, please describe the intervention(s) and why it was abandoned.

____

About retention in care:
The following interventions have been found to be effective in improving retention in HIV care (attending schedule visits). Which of these intervention are currently implemented in you clinic health centre?

	Yes	No	Don't Know
Intervention 1	◯	◯	◯
Intervention 2	◯	◯	◯
Intevention 3	◯	◯	◯

Which of the following reasons may explain why this intervention is not implemented?

- ☐ I am not aware of this intervention
- ☐ Most patients are not eligible for this intervention
- ☐ It will be hard to recruit patient to take part in the intervention
- ☐ My clinic/ health centre is not the right setting for this intervention
- ☐ We don't have the expertise and resources (space, money equipment, etc.,) to deliver this intervention
- ☐ We might have to modify the delivery of the intervention to be able to us it
- ☐ We might have to modify the intervention to ensure that patients are compliant
- ☐ It requires very close follow up
- ☐ The outcome of this intervention is or relevant to participants
- ☐ It doesn't reflect usual care
- ☐ Other reasons (please explain below)

Do you implement any interventions to improve retention in HIV care in your clinic that are not mentioned above?

◯ Yes ◯ No ◯ Don't Know

If your answer was yes, describe the intervention(s):

____

If other, please explain

____

Are there any interventions to improve retention in HIV care that have been tried and abandoned in your clinic/ health centre

◯ Yes ◯ No ◯ Don't Know

If yes, please describe the intervention(s) and why it was abandoned.

____

About You

Which gender do you identify as?	◯ Female<br>◯ Male<br>◯ Non-binary<br>◯ Cis<br>◯ Trans<br>◯ Other
If other, please name what gender you identify as.	__________________________________
What is your age?	__________________________________
Can we contact you again for an interview to discuss the barriers and facilitators to implementing interventions that improve adherence to HIV medication and retention in care? This interview can be by phone or in person.	◯ Yes ◯ No
Phone number	__________________________________
Email:	__________________________________
Best time of day to reach you:	__________________________________
Would you prefer an in-person interview?	__________________________________
