# Supplementary material for: Barriers and facilitators to improving the cascade of HIV care in Ontario: a mixed method study
Source: BMC Health Serv Res. 2024 Jan 10;24:48. doi: 10.1186/s12913-023-10481-z (PMC10782539; doi:10.1186/s12913-023-10481-z)
Supplement: Supplementary file 2 — Additional file 2. PLH Sample Responses. [file 12913_2023_10481_MOESM2_ESM.docx]

**Supplementary file**

**PLH Sample Responses**

| **Theoretical Domains** | **PLH Sample Responses** |
| --- | --- |
| **Skills**  Taking Medication  Peer support    Navigating Care Pathway | “Now it’s just one pill. I just pop it in. Before it was two or three pills.”  “I have plenty of soft skills too, for example, like I said, a peer-to-peer conversation, I didn’t know that I would be able to connect and reach someone”  “And how you will, you know, is this a doctor that’s available that you can speak to a nurse. A nurse can give you a whole guideline. And then if they did refer you to a doctor that is available…” |
| **Knowledge**  Lack of Awareness  Unaware of Cascade of Care prior to Enrolment | “I was ignorant about all this, like literally, they were the ones who [saved] me, because I didn’t know what I was going to do.”  “I didn’t know much about it but when I joined I really got to know more about it. When I came back I started hearing about it.”  “I wasn’t [aware] before I started the cascade of care.” |
| **Beliefs about capabilities**  Capacity to Comply | “Yes. Getting access to medication...my pharmacy delivers my treatment to my door…”  “If I do miss it’s just a day or two.”  “No, no, no I have the ability to do it.” |
| **Professional role and identity**  A “good fit” to the Cascade of Care | “I would say I am the right person. I speak both English and French and that makes it easy. I am very caring.”  “…I know what I want, I know how my body feels, and I know how I need to be treated…”  “Yes, I am. I believe that, I mean in this specific [situation] everyone has a place…” |
| **Memory, attention & decision processes**  Collaborative Decision-making  Proactive Planning | “I think for me it’s all about communication. I’m looking at communication and the more the doctor communicates to me about what’s happening and how to go about it and what options I have it gives me more, a better strategy for how to work for myself and to also follow the cascade.”  “If some day I need it, then I will, you know, start to plan for them in advance, you know, so you start to plan for them.” |
| **Beliefs about consequences**  Cascade of Care “working” for PLH  Needs to Address Stigma | “Yes. I feel it’s working for me.”  “Yes. They worked for me”  “Oh yes, absolutely…even practising my breathing, that even, like I don’t know, the more I did it with the program, the more I got comfortable and the more that I was able to use it in different circumstances.”  “I honestly feel like it works and that’s why I am around. I feel like there is more work to be done. Addressing stigma. For people who use drugs. Stigma is still around. Stigma especially for communities with people of colour.” |
| **Motivations & Goals**  Self-motivated  Staying alive  Support from health care provider  Helping the Community | “You know and motivation is what drives me a lot, but I have to get things done. And how it gets done, I’m very, very self-motivated.”  “I don’t want to die.”  “My personal motivation is if what I’m requesting for is being adhered to or the doctor is being able to look at my concerns then that gives me the motivation to know that OK I think they’re helping with me.”  “For me the motivation is I get to learn and help people who came to Canada”  “I push for everything because I feel this is my health, I need help from the doctors but still if the doctor does not know how I feel internally without me telling them how I feel and insisting on one thing what I want.” |
| **Nature of behaviour**  Learning  Adapting | “I always try to learn what is happening. The new development when it comes, what is new in the community. It facilitates my learning and I am willing to be up to date with developments in science.”  “I think that to me that, you know, there is people as they are not still willing to listen and who are still willing to work with clients and [meet] them where they want to be.” (virtual visits) |
| **Behavioural regulation**  Able to Resist  Does not Resist | “Yes, I can resist, but it’s kind of hard, but I can resist, because, but it’s kind of hard. So, when you see, well OK, so when you see people who you respect, they are people who are depending on you exclusively...”  “No, I [do] what they [health care providers] want and what they say.” |
| **Emotion**  Confidence  Satisfaction  Discomfort | “I feel, I am going to say, I am 80% confident about the interventions. The 20%...unless more is done to educate the community, do more workshops about medications which have changed a lot…the learning (needs to be updated).”  “Yeah I feel I like them [interventions]…”  “I am not used to it.” |
| **Social influences**  Peer Compliance  Mixed Reception | “The people I have spoken to are compliant now. It’s because of the worker and the specialist or the nurse.”  “No. Just my mom. My mom is more (adherent) with medication. I guess she is used to it.”  “I think they do, not all of them of course. Some are not happy some are happy…” |
| **Environmental and context resources**  Insufficient Resources  Community networks  Patient-centred care | “To be honest my friend, there are, I don’t think there was, have ever been enough, I don’t think it has ever been enough. And the reasons why I say that is…because we’re humans, humans have different experiences, right, and [our] experiences are really different. And I think resources are never enough…”  “A strong network. A strong community network….”  “What is missing? Address people struggling with mental health issues, substance use, all of the above. That is what is missing.” |

**Health Worker Sample Responses**

| **Theoretical Domains** | **Health Worker Sample Responses** |
| --- | --- |
| **Skills**  Confidence in Skills      Interpersonal Skills  Integrative Care  Therapeutic Skills | “Yeah so, you know, that’s what I’ve really been doing for the last 30 plus years. I’ve been, you know initiating therapy, and I’ve been follow-up people. So, in terms of like say the initiation part I’m entirely comfortable with that’s involved there.”    “Interpersonal skills to navigate their psychosocial aspects of the need for the intervention. Or even just you know, not to offend people but you know, to understand that people have needs but don't always want to have it pointed out that they have needs. So good interpersonal skills, good communication skills, a [trusting] relationship with the family so that they do feel comfortable to reach out to me when they have needs. So building capacity within relationships. “  “I’ve been in HIV for 30 years and we have a whole team here. So I have a social worker, I have a pharmacist, I have about 10 doctors, so we’re all working as a team and we will do everything possible for the patient, so it’s very easy.”  “And I know that they know so I think in part a very strong therapeutic relationship, a therapeutic relationship is also very important for ensuring that patients are comfortable taking their medications…and their viral loads like have got to be good. So that part of it is fine.” |
| **Knowledge**  Strong Knowledge Base  Need to keep up-to-date | “Yeah so, you know, that’s what I’ve really been doing for the last 30 plus years. I’ve been, you know initiating therapy, and I’ve been follow-up people. So, in terms of like say the initiation part I’m entirely comfortable with what’s involved there. And as part of a team we’re able to do this like show good drug coverage and those types of things which sometimes can be problem. And I’ve definitely got the knowledge like options, drugs and potential side effects and those types of things…”  “I suppose I have a theoretical knowledge about them. I’m not sure how in-depth and the trouble is I keep forgetting what the interventions are so you’ll have to keep reminding me.”  “I have a strong knowledge base about our interventions but I do so over the last few years I have not done as much one-on-one patient education because we have hired another nurse into the clinic, so I don’t engage in this, in like the teaching of medications. So now I would say that my knowledge is not as good as it once used to be.” |
| **Beliefs about capabilities**  Ability to work within a team  Capable of Providing Care | “Yeah, we have the ability to work with the physician.”    “Yeah, or I’d be doing as much as I can to get somebody here”  “So yes, I do think – so I don’t start medications but I certainly support adherence practices.” |
| **Professional role and identity**  Overburdened  Capable of Carrying out Interventions | “There are some of these interventions that I shouldn’t have to do.”  “It is a lot of responsibility and some of the doctors are like, you know, they see the patient in the room and then the rest of it – you know, they prescribe the medication and that's where it ends. So, I would love if we had some of our physicians who wanted to kind of dive in and help with those kinds of things. But it primarily becomes my job (to do extra work).”  “So that – you know as a physician your ability to use some of these things is extraordinarily limited. And without an infrastructure to do anything like that you’re sort of stuck giving some counselling at your appointment time arranging for follow-up appointments in a timely way either with yourself or somebody else. But it’s a challenge knowing what more you as a physician can actually do.”  “I do (have capacity to carry out intervention), although I will tell you, like, it would be nice sometimes to not (be able to perform multiple roles)” |
| **Memory, attention & decision processes**  Heavy workload  Different Capacities depending on health care provider | “My typical day is a nightmare.”  “So, we used to have team meetings but we do not anymore as things got really hectic and our staffing numbers went down and so we were just basically functioning on a daily basis, day-to-day basis. So currently I am not involved in much in the clinical decision-making.”  “I think we have the power to – I mean we always have the ability to suggest an alternate regimen that would fulfil the financial – to decrease the financial burden on somebody, so to be able to get the medication. So, we have that knowledge base to be able to recommend alternative regimens, but we don’t have the power as a pharmacist to implement it without approval from a prescriber.” |
| **Beliefs about consequences**  Setting realistic expectations  Invested Patients and Health Care Providers | “Wanting the best for everybody, but knowing that not everybody needs the same amount of support to have their best outcome.”  “If the patient is not interested it doesn't matter really what we come up with. But if the patient's interested in care and they're collaborating with their health team and then our interprofessional collaborates quite a lot between the doctors, nurses, social workers, support works, to kind of give them that holistic wrap-around care. And so persistence, sometimes. Being creative. And having enough funding to be able to support them.”  “As I said, I think for me it’s more what are the barriers for people and then trying to fix those barriers and I think we either need to meet with them prior going to clinic or a community worker who’s very involved with the clinic that we can, you know – I find we [unintelligible] limitations of our system if that makes sense.” |
| **Reinforcement**  Building relationships  Promoting Health Equity | “I came to this job where everyone has a story. My family is all police officers. For me personally I have a trusting relationship with clients and built trust and it is impactful.”  “The emerging belief that everybody has a right to health and wanting families or patients to – or clients to have the ultimate – you know, have the best potential future for themselves and – yeah, so it's wanting the best for all of my patients and trying to – you know, not everybody needs as much help so you can invest more time with those that do need help and so just- you know.” |
| **Optimism**  Positive Results  Need for Comprehensive Medication Coverage | “So – and I have been in clinic for several, many years and I have seen how beneficial medications are to patients, patients who come in quite sick and diagnosed HIV for the first time, When they start on medications they regain their energy, you know some of their skin issues resolve, they start thriving a little bit more, they’re able to eat properly, you know, they gain a little bit of weight, they feel more energetic.”  “Do I think things are going to improve? No, I think – well for my piece, for instance med access, until someone thinks that there should be no financial burden for someone that needs antiretroviral therapy, I think no, things will not improve and will continue this way.” |
| **Intentions**  Hierarchy of decision-making  Collaborative Practices | “It’s funny to say I'm, personally I feel like I can make those decisions…but there’s also why a lot of times I hold back is because there is usually a hierarchy of kind of a leader, management, so on who are supposed to be making this decision, this final decision. But because of the lack of the knowledge, they don’t make it.”  “We have a very collaborative group practice. I mean like all practices in Canada it’s not huge. The challenge is that we were within a hospital setting so there is a limitation to what we can try to implement when it involves new infrastructure that’s needed.” |
| **Goals**  Optimize Patient Health  Passionate about work | “Ensure or to optimize patients' health, right? And keep them out of the hospital so by keeping them on their meds they can stay healthy. And hopefully stay out of the hospital. And you know, we're unique compared to most of the other clinics because I'm, you know, I deal with paediatric patients, right?”  “Yeah, my ultimate goals for our patients are [they’re] physically well, mentally well and looked after holistically. So you know, if someone has no access to nutritious food then I want to get them on our food program.”  “So my goals are to improve patient health, so basically what I’m doing – or improve patient health and knowledge and so through patient education I’m hoping that patients will glean some sort of knowledge through our discussions about whether it be STDs or have them better adhere to their medications in order to, you know, help support their own bodily functioning to improve.”  “I'm extremely passionate about my work and passionate about getting folks care and on treatment so that they can live long healthy lives.” |
| **Behavioural regulation**  Ability to resist | “So, do I have agency to resist doing that? Yes. And then I would have agency to implement my own bias, you know, that's not how I care for my patients. I try to treat everybody equitably and – yeah.” |
| **Emotion**  Thinking Positively  Worry for the future of HIV care | “I think it’s still very positive. I think that a lot, there’s always sometimes like a little bit of with once it becomes very popular…”  “They [new hires] don’t have the same knowledge, OK. What I find is that it’s the transfer of knowledge and the other services... so that’s going to be an issue. My social worker will retire soon, and I have new staff and they don’t have quite the same knowledge and I find that’s where we need *to do something and that’s the biggest fear for HIV future.”* |
| **Social influences**  Shared vision among peers  Dedicated staff | “What their belief is about them, I think would be the same. We're all striving to keep our patients as healthy as possible so that they can live to their fullest – within their capacity to do so. I think, you know, a social worker might use more of a psychosocial approach as well to – you know, where I don't a have that luxury of doing counselling with them, you know, long-term counselling with them or any kind of lengthy counselling.”  “I think we have a stellar team here. And I rely heavily on our main lead and our support workers and the other nurses to ensure that folks get looked after. But what I'm grateful is I've managed to hire really passionate folks…” |
| **Environmental and context resources**  Challenges with Access  Insufficient resources | “It’s the reengaging them that is sometimes difficult because they may live a distance from Kingston and not have cellphone access. And, you know, we do work with our community partners quite a bit including the local HIV/AIDS resources but that’s limited sometimes because not all of our patients access them.”  “We do not have adequate funding for nursing. We do not have access to – or rapid access to psychiatry. We do not have a therapist. I end up doing psychotherapy here because I'm he only one that's really qualified…” |
